# Prevalence and drug resistance of mycobacteria in Turkish cystic fibrosis patients

**DOI:** 10.1186/1476-0711-13-28

**Published:** 2014-08-13

**Authors:** Dilek Satana, Gonca Erkose-Genc, Zeynep Tamay, Meltem Uzun, Nermin Guler, Zayre Erturan

**Affiliations:** 1 Istanbul Faculty of Medicine, Department of Medical Microbiology, Istanbul University, Istanbul 34093, Turkey; 2 Istanbul Faculty of Medicine, Department of Pediatrics, Istanbul University, Istanbul 34093, Turkey

**Keywords:** Mycobacteria, Nontuberculous mycobacteria, *Mycobacterium tuberculosis* complex, Cystic fibrosis, Drug resistance

## Abstract

**Background:**

Isolation of mycobacteria in cystic fibrosis (CF) patients is increasingly being reported. Because of having long term antimicrobial treatment, CF patients are at risk of pulmonary infection with especially resistant nontuberculous mycobacteria (NTM) strains. The aim of the present study is to determine the prevalence of *mycobacterium* spp. and antimicrobial susceptibility in Turkish CF patients.

**Methods:**

During a 5.5 year study period, 376 sputa from 130 CF patients were analyzed. Antimycobacterial susceptibility testing was performed by the Bactec 460 TB System and the E test method.

**Results:**

Totaly 28 (7.44%) *Mycobacterium* spp. were isolated from eight (6.15%) CF patients. Five isolates (17.9%) were identified as *Mycobacterium tuberculosis* complex (MTBC), 14 (50%) as *Mycobacterium abscessus* and nine (32.1%) as *Mycobacterium lentiflavum*. All MTBC isolates were found to be susceptible to streptomycin, isoniazid, rifampicin, and ethambutol. Resistance to some antibiotics was detected in some NTM strains. These are the first data about the prevalence of mycobacteria in CF patients from Turkey.

**Conclusions:**

In pediatric CF patients, specific mycobacterial analysis of sputum specimens and susceptibility testing should be performed for allowing early detection, identification and the possibility of eradication of these bacteria.

## Background

Cystic fibrosis (CF) is an autosomal recessive disease which defects in the Cystic Fibrosis Transmembrane Conductance Regulator (CFTR) gene product result in abnormally viscous secretions, mucus plugging of the airways, intense inflammation, chronic airway infection, and early death due to progressive bronchiectatic lung disease [1].

Infection with *Mycobacterium tuberculosis* complex (MTBC) strains in this patient group is rarely reported (1.34-3.41%) [2-8]. These patients are at an increased risk of pulmonary colonization with opportunistic microorganisms. Nontuberculous mycobacteria (NTM) are increasingly isolated from patients with CF, although the clinical significance of NTM in this population is not yet entirely understood [1]. It is not known whether these organisms are transmitted from person to person, acquired from environmental sources or obtained nosocomial [9]. Person-to-person transmission of NTM has been considered unlikely. However Aitken et al. [10] recently reported an outbreak of *Mycobacterium abscessus* ss *massiliense* in five cases with CF. The underlying structural airway disease and altered mucociliary clearance may be the reasons for the high prevalence of NTM in this patient group. Potential risk factors for NTM colonization/infection in CF include steroid treatment of allergic broncho-pulmonary aspergillosis and the use of bronchoscopes, aerosolized medications and tap water contaminated with NTM [9].

Multiple centers all over the world have reported a prevalence ranging from 1.09% to 22.7% for NTM in respiratory specimens of CF patients [3,6,7,11-16]. Oliver et al. [11] prospectively studied 986 CF patients between 10–51 years of age and found a prevalence of 13%. The author reported that *Mycobacterium avium* complex (72%) and *Mycobacterium abscessus* (16%) were the most common species. An increased isolation rate of *M. abscessus* from CF patients has been reported in several countries [6,11,13-15,17,18]. The American Thoracic Society (ATS) published guidelines about the diagnosis and treatment of lung disease caused by NTM [9]. To meet criteria for NTM infection, patients must have compatible clinical findings, compatible radiographic findings, and bacteriologic findings including three positive cultures or two positive cultures and a positive smear for acid-fast bacteria. The first two criteria have been difficult to apply to patients with CF because of their longstanding respiratory symptoms, infective exacerbations, pre-existing radiographic abnormalities and systemic symptoms [3,9]. The ATS microbiologic criteria related to this subject, require the following; 1. Positive culture results from at least two separate sputum samples or 2. Positive culture result from at least one bronchial wash or lavage or 3. One or more sputum or bronchial washings that are culture positive for NTM if mycobacterial histopathologic features were evident [9].

Because of the risk of increased resistance due to long term antimicrobial treatment of common CF pathogens, susceptibility testing is recommended for NTM isolates from these patients [19]. *M. abscessus*, which is one of the most often isolated mycobacteria from CF patients, is reported to be resistant to most of the antimycobacterials including tetracyclines, fluoroquinolones, and sulphonamide [20,21]. Mussaffi et al. [13] reported six CF patients with NTM pulmonary disease, five of them had positive cultures for *M. abscessus* and one had a positive culture for *M. simiae*. All six cases were infected with multiresistant strains. The researchers observed that even when the NTM was susceptible initially, resistance developed rapidly after institution of therapy. In another study, *M. chelonei* and *M. fortuitum* were isolated from sputa of two different CF patients and found to be resistant to all drugs tested [3].

There is only two reports about multidrug resistant tuberculosis in this patient group. Two *M. tuberculosis* strains which were isolated from two patients with pulmonary infection, found to be resistant to all antibiotics [2,8].

The prevalence of mycobacteria in Turkish CF patients is not known. The aim of the present study is to reveal the prevalence of MTBC and NTM isolates in Turkish CF patients and to determine the antimicrobial susceptibility of isolated species.

## Methods

During a 5.5 year study period from April 2003 until November 2008, sputa which were sent to the Istanbul Faculty of Medicine, Department of Medical Microbiology for routine bacteriological or specific mycobacteriological culture were analyzed. The approval of an ethics committee was not necessary, because the clinical samples of the study were taken as part of standard patient care. Informed consent was given by all patients in this study. During this period a total of 376 respiratory specimens were collected from 130 patients.

Specimens were processed according to the standard protocols [22]. All sputa were decontaminated by N-acetyl-L cysteine (NALC)/ sodium hydroxide (NaOH) and concentrated by centrifugation (3800 g for 15 min at +4).

Processed specimens were prepared and stained by the Ziehl-Neelsen (ZN) method. The sediment was resuspended in 1 ml phosphate buffer (pH 6.8) and 0.4 ml was inoculated into Bactec 12B medium supplemented with antibiotics (Becton Dickinson, Spark, MD, USA) and onto Lowenstein-Jensen medium (Becton Dickinson, Spark, MD, USA). All cultures were incubated at 37°C for 10 weeks. The slants were examined twice weekly for two weeks and then weekly for a further 8 weeks. The Bactec 460 TB vials were controlled every 2 days during the first week and weekly thereafter.

ZN stained smears were prepared from positive Bactec 460 TB vials or LJ cultures. All acid-fast bacilli were identified to species level by the GenoType Mycobacterium CM/AS (Hain Lifescience GmbH, Nehren, Germany) assay.

Antimycobacterial susceptibility testing for MTBC isolates were performed by the Bactec 460 TB System. The critical concentrations used for the determination of resistance to major antimycobacterial agents were as follows: rifampicin 2 μg/ml, isoniazid 0.1 μg/ml, ethambutol 2.5 μg/ml, and streptomycin 2 μg/ml [22].

Susceptibility testing for NTM isolates was performed by the E test method according to the manufacturer’s instructions to clarithromycin, tigecycline, linezolid, amikacin, trimethoprim-sulfamethoxazole, doxycycline, cefoxitin, ciprofloxacin, imipenem, and tobramycin. The MIC values were evaluated according to the Clinical and Laboratory Standards Institute. The reference strain *Staphylococcus aureus* ATCC 29213 was used for E test, and H37Rv ATCC 27294 was used for the Bactec 460 TB System as quality control strains.

## Results

A total of 376 sputa from 130 CF patients were investigated over a period of 5.5 years. The mean age of the patients was 12.1 ± 3.1 years (range: 5–17). Out of 376 samples, a total of 28 (7.44%) *Mycobacterium* spp. were isolated from eight (6.15%) CF patients. Characteristics, culture and smear results of *Mycobacterium* spp. positive patients are given in Table 1. Five isolates (17.9%) were identified as MTBC, 14 (50%) as *M. abscessus* and nine (32.1%) as *M. lentiflavum*. The isolation rate according to the patients was 3.07% (4/130) for MTBC and NTM strains each. Five MTBC strains were isolated from four different patients (two times from one patient), 14 *M. abscessus* strains were isolated from three different patients at different times, and nine *M. lentiflavum* strains were isolated from the same patient at different times. Three of four (75%) NTM positive and one of four (25%) MTBC positive patients were also smear positive. The contamination rate of cultures was 9.8% (37/376). Co-pathogens isolated together with *Mycobacterium* spp. were *Pseudomonas aeruginosa* (n = 5), *S. aureus* (n = 4), *Stenotrophomonas maltophilia* (n = 4), and *Aspergillus fumigatus* (n = 2). The characteristics of all patients, smear results and the distribution of co-colonizing bacteria are given in Table 2.

**Table 1:** **Characteristics, culture and smear results of *Mycobacterium *** **spp. positive patients**

**Patient**	**Sex**	**Smear (ZN)**	**Culture results**	
			***Mycobacterium *** **spp. (n)**	**Co-pathogens**
1	M	+	MTBC (2)	*Staphylococcus aureus*
2	F	-	MTBC (1)	*Stenotrophomonas maltophilia, Staphylococcus aureus, Pseudomonas aeruginosa*
3	M	-	MTBC (1)	*Stenotrophomonas maltophilia, Escherichia coli, Enterobacter spp*.
4	F	-	MTBC (1)	Not detected
5	M	+	*M. lentiflavum* (9)	*Stenotrophomonas maltophilia, Staphylococcus aureus, Pseudomonas aeruginosa, Aspergillus fumigatus*
6	F	+	*M. abscessus* (10)	*Stenotrophomonas maltophilia, Pseudomonas aeruginosa, Aspergillus fumigatus*
7	F	+	*M. abscessus* (3)	*Pseudomonas aeruginosa*
8	F	-	*M. abscessus* (1)	*Pseudomonas aeruginosa*

M: Male, F: Female, MTBC: *Mycobacterium tuberculosis* complex.

**Table 2:** The characteristics of patients and the distribution of co-colonizing bacteria

**Characteristic**	**MTBC and NTM (-)**	**MTBC (+)**	**NTM (+)**
Total (n = 130)	122	4	4
Female (%)	58 (47.5)	2 (50)	3 (75)
Mean age (years)	12.1 ± 3.2	11.5 ± 1.2	15.5 ± 1.7
*Pseudomonas aeruginosa* (%)	26.51	-	100
*Staphylococcus aureus* (%)	28.03	50	50
*Stenotrophomonas maltophilia* (%)	10.10	50	50
*Haemophilus influenza* (%)	8.83	-	-
*Aspergillus* species (%)	8.08	-	50
*Streptococcus pneumonia*e (%)	4.54	-	-
*Klebsiella pneumonia* (%)	1.51	-	-

All MTBC isolates were found to be susceptible to streptomycin, isoniazid, rifampicin, and ethambutol.

All *M. abscessus* isolates were susceptible to clarithromycin (MICs 0.125-1 μg/ml) and tigecycline (MICs 0.19 -0.5 μg/ml). Eleven (78.6%) of 14 strains were determined as susceptible to linezolid (MICs 4 μg/ml), while the remaining three (21.4%) were resistant (MICs >256 μg/ml). One strain (7.1%) was found to be resistant to amikacin (MIC > 256 μg/ml) and 13 strains (92.8%) showed intermediate MICs (32–48 μg/ml). All *M. abscessus* isolates were resistant to trimethoprim-sulfamethoxazole (MICs > 32 μg/ml), doxycycline (MICs >256 μg/ml), cefoxitin (MICs >256 μg/ml), ciprofloxacin (MICs >32 μg/ml), imipenem (MICs >32 μg/ml) and tobramycin (MICs >256-64 μg/ml).

All of the *M. lentiflavum* isolates were susceptible to clarithromycin (MICs 0.25 μg/ml), amikacin (MICs 12 μg/ml) and ciprofloxacin (MICs 0.032 μg/ml), and resistant to streptomycin (2 μg/ml), isoniazid (0.1 μg/ml), rifampicin (2 μg/ml), ethambutol (2.5 μg/ml), imipenem (MICs >32 μg/ml), tigecycline (MICs > 256 μg/ml), cefoxitin (MICs >256 μg/ml), doxycycline (MICs 128 μg/ml), and tobramycin (MICs 64 μg/ml).

## Discussion

There is little information about pulmonary tuberculosis in CF patients, and only a few cases have been published [2-8]. The isolation rates of MTBC from respiratory samples of these patients were reported as 1.34-3.41% [3,6,7].

These are the first data about the prevalence of mycobacteria in CF patients from Turkey. In our study, MTBC was isolated from sputa of four out of 130 patients (3.07%). This rate is similar to the results reported from other studies.

NTM have emerged as new pathogens in CF patients recently [23]. CF centers worldwide have reported isolation rates ranging from 1.09% to 22.7% [7,12,13,15,16,19,24-27].

We found that during the study period 3.07% (4/130) of Turkish CF patients had NTM in their sputum samples. Three of these patients (75%) had positive smears, and met the ATS microbiologic criteria [9].

Recovery of NTM from sputa of CF patients is usually hampered by the presence of other bacteria, especially *P. aeruginosa*, which is able to survive after routine sputum decontamination using NALC-NaOH. The Centers for Disease Control and Prevention recommend 5% oxalic acid (OxA) as decontaminating agent for CF specimens [28]. Whittier at al. [29] showed that using 5% OxA after NALC-NaOH resulted with decrease in contamination rates and increase in recovery of mycobacteria. However Bange et al. [30] reported that although the contamination rate was reduced with NALC-NaOH-OxA, the overall sensitivity of mycobacterial recovery remained the same. In addition, Radhakrishnan et al. [19] pointed out that decontamination with NALC-NaOH-OxA may be too harsh and eliminate NTM in smear negative samples with lower numbers of mycobacteria. In an multicenter investigation Whittier et al. [28] concluded that although with the NALC-NaOH-OxA method NTM were successfully recovered from samples with AFB smear scores of 3+/4+, but this was problematic in low inoculum samples with AFB smear scores of 1+/2+. In the current study, only NALC-NaOH was used as decontaminating agents. Three of four (75%) NTM positive and one of four (25%) MTBC positive samples were smear positive with scores of 1+/2+. Using OxA could have resulted with decrease in recovery rates of mycobacteria.

*M. abscessus* is a rapid growing mycobacterium (RGM) which is reported as a causative agent of respiratory disease in patients both with and without predisposing conditions, and cutaneous disease usually following accidental trauma or surgery [9]. It is one of the NTM that are most commonly isolated from CF patients, especially at younger ages [9,13,20]. *M. abscessus* isolates are uniformly resistant to standard antituberculous agents and infection with these microorganisms is extremely difficult to treat. Antibiotic susceptibility testing of clinically significant isolates is recommended due to variable in vitro drug susceptibilities to some drugs. Acquired mutational resistance to clarithromycin and amikacin can occur [9,31].

The isolation rate of *M. abscessus* from pulmonary samples of CF patients was reported as 1.1-14.1% [11-14,25,26]. Reported rates of pulmonary infection with these species according to the ATS microbiologic criteria was 1.5-3.8% [13,15,17,24,27].

In the current study, *M. abscessus* was the most commonly isolated NTM species (60.9% of NTM species). *M. abscessus* was isolated from three patients with an isolation rate of 2.3%, and two of these patients (1.5%) met the ATS microbiologic criteria (positive culture results from at least two separate sputum samples) [9]. Although the isolation rate of this species from sputa of Turkish CF patients is low, it is in concordance with results reported from other studies.

*M. lentiflavum* is placed between the most commonly isolated newly described NTM species from clinical samples [32]. It was reported as the agent of cervical lymphadenitis mostly in children, skin and invasive infections, and a few respiratory tract infections have been detected in both immunocompetent and immunocompromised patients [32-34]. Because of its low virulence, the isolation of this species does not usually refer to an infection [32]. Immunosupression is a risk factor for infection with *M. lentiflavum*[33]. Resistance to primer antituberculous drugs is commonly reported [32-35].

There are a few studies which reported the isolation of *M. lentiflavum* from respiratory samples of CF patients with a rate of 0.06-20% [6,11,15,17]. However, none of these studies reported a case which meets the ATS criteria for pulmonary infection.

In this study, *M. lentiflavum* was isolated from one patient’s (0.8%) nine separate samples, and this patient met the ATS microbiologic criteria. *M. lentiflavum* was consisted of 39.1% of NTM species isolated in the current study. The isolation rate of this species is similar to the rate which was reported in the other studies.

Oliver et al. [11] compared CF patients with and without NTM and found that culture-positive subjects had a higher frequency of *S. aureus* and a lower frequency of *P. aeruginosa*. They hypothesized that the presence of *S. aureus* may create conditions which can favor the presence of NTM in the airways. However Levy et al. [24] noted that there was no difference among the isolation rates of *S. aureus* from culture positive and negative patients regarding NTM. Interestingly, Musaffi et al. [13] and Levy et al. [24] reported that CF patients with NTM colonization and lung disease had higher frequency of *P. aeruginosa*. The same was observed in the current study. The rate of NTM and *S. aureus* co-colonization was 21.7%, whereas the rate of NTM and *P. aeruginosa* co-colonization was higher as 34.7%.

Long term use of macrolide in CF patients was shown to be a risk factor for NTM infection, especially with *M. abscessus*[24]. Renna et al. [36] showed that concentrations of azithromycin inhibited intracellular killing of mycobacteria in macrophage by impairing autophagic and phagosomal degradation and resulted in chronic infection in a mouse model. However, as there are some studies which have not established any association [19,26,37,38], the link between chronic macrolide use and NTM infection remains indeterminate.

In this study nine of the patients had long term macrolide therapy. Two of them (22.2%) had infection with *M. abscessus*, one (11.1%) had infection with *M. lentiflavum*, and one (11.1%) other patient had infection with MTBC. As number of patients using macrolide is very small, the relationship between macrolide use and NTM infection could not get analyzed statistically.

In CF patients there is often poor correlation between in vitro drug susceptibility testing and clinical response for most antimicrobial drugs except for the macrolides. For *Mycobacterium avium* complex, susceptibility testing to macrolides is recommended because of the correlation with clinical response. Macrolide sensitivity testing and identification of the exact subspecies of *M. abscessus* also provides useful information for the treatment. Because of a deletion in the inducible erythromycin ribosome methyltransferase (*erm*) gene, *M. abscessus* subspecies *massiliense* is susceptible to macrolides, while *M. abscessus* ss *abscessus* and *M. abscessus* ss *bolletii* is resistant [39,40].

There are only three studies which reported the drug susceptibility of MTBC strains which were isolated from CF patients. Asherova et al. [2] isolated two MTBC strains from two CF patients with pulmonary infection and found out that the two strains were resistant to all antibiotics tested. Smith et al. [3] isolated MTBC from sputa of three CF patients and reported that these three strains were susceptible to streptomycin, isoniazid, rifampicin, ethambutol, p-aminosalicylic acid, ethionamide, cycloserine, capreomycin, and kanamycin. Two of these strains were susceptible to pyrazinamide, while one strain was resistant. In the case report by Manika et al. [8] the strain was found resistant to streptomycin, isoniazid, rifampicin, ethambutol, pyrazinamide and quinolones, while it was susceptible to amikacin, kanamycin and capreomycin. In the current study all of MTBC isolates were found to be susceptible to streptomycin, isoniazid, rifampicin, and ethambutol.

*M. abscessus* is one of the NTM species which is most commonly isolated from CF patients and known to be the most pathogenic and chemotherapy-resistant RGM [9,13,20]. However there are only a few reports about the drug resistance of strains isolated from CF patients. Sanguinetti et al. [21] isolated *M. abscessus* from a CF patient which developed fatal disseminated infection. This strain was found to be resistant to streptomycin, isoniazid, rifampicin, ceftazidime, clarithromycin, levofloxacin, vancomycin, imipenem, amoxicillin, ethionamide, trimethoprim-sulfamethoxazole, ofloxacin, and amoxicillin-clavulanic acid. In another study, *M. abscessus*, which was the causative agent of a pulmonary infection in a CF patient, was initially susceptible to clarithromycin, ethambutol, amikacin, mefoxitin and developed in vitro resistance to all these drugs except for ethambutol after therapy [41]. Jönsson et al. [15] recovered a *M. abscessus* strain from a CF patient with persistent airway colonization, and detected that it was resistant to rifabutin, clarithromycin, clofazimine, and amikacin. In the current study all of the *M. abscessus* isolates were susceptible to clarithromycin and tigecycline. Eleven (78.6%) of 14 strains were determined as susceptible to linezolid, while the remaining three (21.4%) were resistant. One strain (7.1%) was found to be resistant to amikacin and 13 strains (92.9%) showed intermediate sensitivity pattern. All of *M. abscessus* isolates were resistant to trimethoprim-sulfamethoxazole, doxycycline, cefoxitin, ciprofloxacin, imipenem, and tobramycin.

Although the isolation of *M. lentiflavum* from respiratory samples of CF patients is reported in a few studies, there is no information about the susceptibility pattern of these strains [6,11,15,17]. In studies with *M. lentiflavum* strains isolated from non-CF patients, this species was usually found to be resistant to primary antituberculous drugs [32-35]. However, strains which are susceptible to clarithromycin, amikacin, ciprofloxacin, cycloserin, canamycin, and ofloxacin are reported [32,34,35]. In this study, all of *M. lentiflavum* isolates were susceptible to clarithromycin, amikacin, and ciprofloxacin, while they were fully resistant to streptomycin, isoniazid, rifampicin, ethambutol, imipenem, tigecycline, cefoxitin, doxycycline, and tobramycin.

## Conclusions

Even though infection by MTBC in CF patients is rare, pulmonary infections due to NTM are increasingly being reported [2-4,7,11,14]. These are the first data about the prevalence of mycobacteria in CF patients from Turkey. We recovered MTBC from sputa of these patients with a rate of 3.07%, and the isolation rate of NTM in this study is 3.07%. In pediatric CF patients, specific mycobacterial analysis of sputum specimens are not routinely performed. In our opinion this should be done for allowing early detection, identification and the possibility of eradication of these bacteria. As these patients are under heavily antibiotic treatment, infection with resistant strains may occur. Because we detected resistance to some antibacterials in NTM strains, we propose that susceptibility testing should be performed for the NTM strains isolated.

## Competing interests

The authors declare that no competing interest exists.

## Authors’ contributions

DS performed the experimental work and helped to draft the manuscript. GEG performed the experimental work and drafted the manuscript. ZT and NG carried out the clinical management of the patients. MU participated in the control and evaluation of the experiments. ZE designed the study and analyzed the data. All authors read and approved the final manuscript.
